# There is an urgent need for a global rural health research agenda

**DOI:** 10.11604/pamj.2022.43.147.38189

**Published:** 2022-11-18

**Authors:** Luchuo Engelbert Bain, Oluwafemi Atanda Adeagbo

**Affiliations:** 1 International Development Research Centre (IDRC), Ottawa, Canada,; 2 Department of Psychology, Faculty of Humanities, University of Johannesburg, Auckland Park, South Africa,; 3 Department of Community and Behavioral Health, College of Public Health, University of Iowa, Iowa, United States,; 4 Department of Sociology, University of Johannesburg, Auckland Park, South Africa

**Keywords:** Rural, health, global, research, agenda

## Abstract

People living in rural areas generally experience adverse health outcomes compared to their urban counterparts. They experience a greater burden of non-communicable diseases including: diabetes, hypertension, stroke, kidney disease, and chronic obstructive pulmonary disease (COPD), have limited access to healthcare services, and experience scarcity in specialized healthcare services. The disproportionately high all-cause mortality experienced by rural residents has been termed “the rural mortality penalty”. With over 90% of the world’s rural population living in Africa and Asia, we argue that the lack of an authoritative and respected global rural health research agenda contributes to increasing health inequalities, given that many of these people are receiving substandard care. There are differences in how rural and urban resident’s experience healthcare. Living in rural settings might not be systematically connected to adverse health outcomes. It is important to clearly articulate the positive health outcomes associated with living in rural settings (e.g., the positive relationship between mental health and strong social ties/green spaces). Indeed, health policies stand the chance of unconsciously excluding the positive outcomes associated with rurality, as well as the rural experiences of health. Defining rural health remains an issue of controversy with a persistent reality regarding the lack of consensus as to what it means for a region or area to be considered as “rural”. We outline the most common definitions of “rural areas” in the literature, as well as the shortcomings of these definitions. By unpacking the meaning of “rural health”, we aim to foster communication among rural health professionals and researchers locally and internationally, as well as highlight the key research and policy implications that could emanate from a “good” definition of rural health. We agree that context remains key when it comes conceptualizing complex subjects like rurality. However, developing minimum criteria to foster communication among rural health researchers is needed. Systematically providing operational definitions of what authors describe as “rural” in the rural health research and policy literature is of utmost relevance.

## Essay

### Introduction

Place and context affect the overall health status of populations [1-3]. People living in rural settings are increasingly reported to experience a health disadvantage and disparities. They are disproportionately prone to negative health outcomes compared to their urban counterparts [1-5]. Rural populations are more likely to have a poorer quality of life, have a relatively greater number of people without health insurance, limited access to quality healthcare services, have fewer healthcare infrastructures, lower socioeconomic status, and lower life expectancy compared to their urban counterparts [1-6]. These discrepancies in mortality between urban and rural residents have been referred to as the “rural mortality penalty” [4]. Of the over 9.7 million deaths recorded in 2017 in India, three quarters occurred in rural areas [5]. Irrespective of their physical environment or socioeconomic status, rural populations often experience higher burdens of diverse conditions such as obesity, diabetes, cancer, severe acute malnutrition, and injury compared to urban populations [3,4,6]. They are also more likely to engage in risky behaviours such as substance abuse [3,4,6].

Further, the recent coronavirus (COVID-19) pandemic has highlighted the vulnerability of rural communities during an outbreak. In the US per capita, disease burden and health system demand are the highest outside of urban areas [7]. Despite clear inequities existing between rural and urban populations, there has been a lack of political will to address these persistent challenges. Empirical research remains sparse in systematically addressing these inequalities. The Sustainable Development Goals (SDGs), which are an urgent call for action by all countries, recognise that ending poverty and other deprivations must go hand-in-hand with strategies to improve health and education, reduce inequality, and spur economic growth [8]. It is estimated that 76% of the developing world's poor live in rural areas [9]. The ability to recruit and retain a sustainable, skilled workforce to address these inequities and facilitate access to community-relevant care in rural and remote regions is dependent upon evidence-based operational research and situational analysis [10].

To achieve SDG 10 of bridging existing inequalities among countries, the intricate connections between health, poverty, and wellbeing and the application of health policies that do not fit the contextual needs of rural communities, must be considered if health policies targeting rural populations do not match their needs. Adequate policies should be informed by evidence. We argue that the global rural health research agenda has not been articulated enough. Indeed, a definition of rural health that meets a minimum consensus is required to allow communication among rural health researchers. Defining rural health remains an issue of controversy [11]. In this paper, we report key issues around rural experience of health, and articulate the need for a rural health research agenda, with issues around an 'inclusive' definition for rural health at its core.

### Rural experiences of health

The rural setting is very important to rural residents. The rural context must not always be seen in the negative sense [12,13]. The social relations between individuals, family ties, and valued community identities have been reported to be very important considerations for rural residents [14,15]. Disrupting these ties or policies that unintentionally disrupt the will of the rural residents can be detrimental to their wellbeing. Access to green spaces, far more common in rural settings, are increasingly being reported to be associated with better mental health outcomes [16]. What it means to be healthy differs from one rural population to the other [11,17,18]. The extremely rural heterogeneity makes one-size fit-all approaches challenging to implement [19,20]. Engaging communities in the co-creation of health intervention, design and implementation should be prioritized in order provide culturally affirming healthcare services that will improve their health outcomes and overall wellbeing [13,15,21]. These approaches are more likely to provide context-specific solutions to rural health concerns. Framing a rural health research and policy agenda requires a deep understanding of how rural residents perceive and experience health. Table 1 summarizes how rural residents perceive and experience health in the literature.

**Table 1:** rural perception and experience of health

Author and manuscript title	Key issues raised
Thurston and Meadows, 2003: rurality and health: perspectives of mid-life women	- 'Rural health’ in a limited sense (only to mean the health of farmers)
	- The rural environments are not just as a setting for research but also as a social construct.
	- Described rural living as very important to their health.
	- Highlight heterogeneity of ‘rurality’ (context, each rural setting is special).
	- The findings challenge assumptions about the detrimental relationship between rurality and health. Researchers and policy makers would be wise to listen to inhabitants to develop contextually relevant research and policy.
Gessert *et al*. 2015: rural definition of health: a systematic literature review	- Findings based on US, Canadian, and Australian populations.
	- Possible that rural residents might have relevant and unique views on what it means to be healthy.
	- Rural community heterogeneity recognized (culture, economic hardship, sense of community/history).
	- If rural and non-rural populations think about or define their health differently, efforts to engage them in promoting and preserving health must be better informed, particularly as healthcare providers increasingly focus on patient-centered care.
	- Rural settings change, important to have indicators of rurality that are more agile (responsive to easily captured changing parameters).
Kenny *et al*. 2013: community participation in rural health: a scoping review	- Highlight the relevance of evidence - informed community participation in rural healthcare.
	- One size fits all approaches are ineffective.
	- Harnessing community capacity is integral to developing locally responsive health services, especially regarding gaining trust and acceptance.
Harvey, 2007: understanding Australian rural women’s ways of achieving health and wellbeing - a metasynthesis of the literature	- The social experiences of rural-women influence the way they construe their health and wellbeing.
	- Heterogeneity, important to study the interactions of social, cultural and economic dimensions.
Rawolle *et al*. 2016: farmers’ perceptions of health in the Riverland region of South Australia: ‘If it’s broke, fix it’	- Participants perceived health as being able to function and complete farm work. Participants described how health was influenced by community activities and social support from friends and families. Healthcare providers should frame interventions to resonate with the perceptions of health held by local communities
Kulig *et al*. 2009: how do registered nurses define rurality?	- The highest frequency of registered nurses responses identified features of the community as central to the definition of rurality, with both positive characteristics (e.g. lifestyle) and challenges (e.g. access to amenities) identified, as well as the importance of the availability of human and technical resources.
	- Although distance to resources was important, the huge variability of absolute distances cited underscores the futility of developing a national numerical index of rurality based on distance to services.

### Why do we need global research agenda for rural health?

In 2018, 3.4 billion (44.7%) of the world´s 7.6 billion inhabitants lived in rural areas, and Africa and Asia are home to 90% of the world´s rural population [22]. There are glaring cultural differences (family, values, relationships, meanings in community) between urban and rural areas. Rural healthcare research and policy are still lagging in these regions of the world [23]. Social and cultural norms have been reported to influence health seeking behaviors [24,25]. Unaddressed rural health challenges might lead to health inequalities. For instance, failure to improve health outcomes in rural areas will indirectly push its residents into the spiral of poverty, ill-health and low economic productivity [26]. In China, dusty lung deaths for men living in rural areas doubled compared to their urban counterparts between 2002 and 2016 [27]. This has been attributed to policies that completely excluded rural communities [27]. Thus, empirical evidence is crucial to make a strong case for rural residents.

**Figure 1:**
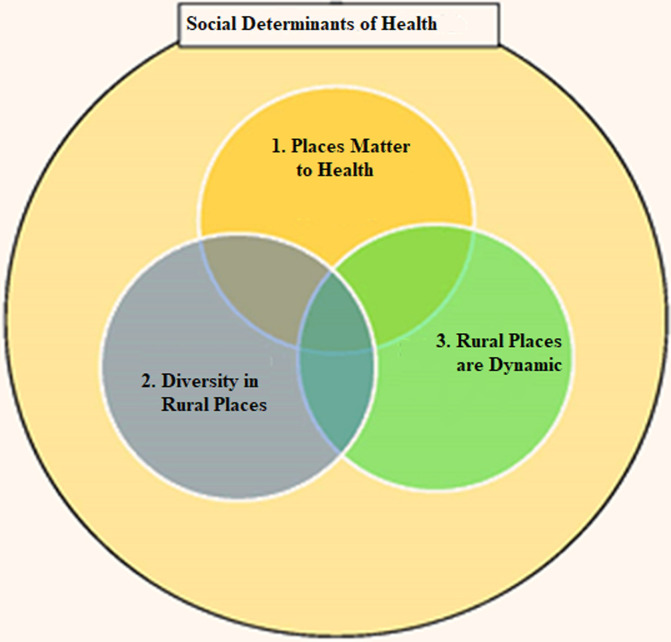
a general rural health research framework

Robust evidence could serve as an important advocacy instrument to bring about change. If rural residents are not properly identified with denominators that capture them appropriately as well as their sociocultural identities, they will be either completely forgotten or at times receive services that do not match their needs and, culture. There are many commonalities as well as differences across rural settings worldwide. However, responding to this challenge needs to be context specific. Responses in land locked areas could differ from responses in areas accessible only by boat, ship, or air. Systematically identifying these differences and commonalities can be helpful in generating local evidence, which could serve as learning grounds for other rural settings. The “rural mortality penalty” is a reality worldwide and has been mostly documented in the US [4,28,29]. There is however a growing body of literature on the heterogeneity in the mortality distribution across rural settings [28,29]. Context and social determinants of health are important considerations when reading mortality rates in rural settings. In 2016, Kulig proposed a rural health framework composed of three core interrelated areas: 'places matter to health', 'diversity in rural places', and 'rural places are dynamic' [30]. She posits that these three areas are interconnected by the social determinants of health (Figure 1). This framework could be empirically tested in diverse rural settings to ascertain its suitability in guiding rural health research and policy.

### in rural health conceptualization and common definitions of rural areas in selected countries

Current perspectives and focus on rural health are fraught with fundamental shortfalls including lack of common definition of rural area and subsequently rural health as well as the weak theoretical framework for implementation of rural health concept. Globally, there is no univocal definition of what area can be considered rural. Most of these definitions have traditionally provided a dichotomous meaning of where to consider rural or urban. In fact, the definitions often differ even within the same country. Population density, travel time to the nearest urban centre, and access to services are mostly used in the definition of rural areas (Table 2).

**Table 2:** examples of common definitions of ‘rural areas’ in selected countries

Country	Defining Body	Definition of rural
**Canada**	Statistics Canada https://www150.statcan.gc.ca/n1/pub/21-006-x/2008008/section/s2-eng.htm	Small towns, villages, and other populated places with less than 1,000 population according to the current census
	Ontario Ministry of Health and Long-term Care (2012) (Ref.: rural and Northern Ireland healthcare framework	An area is rural if it has ‘a population of less than 30,000 [and is] greater than 30 minutes away in travel time from a community with a population of more than 30,000’ (Note: classification on the basis of population density)
**Australia**	Australian Statistical Geography Standard (ASGS)	Remoteness Areas (RAs) divide Australia and the States and Territories into 5 classes of remoteness on the basis of their relative access to services (major cities, inner regional, outer regional, remote, very remote) ‘rural and remote’ generally cover all areas outside Australia’s major cities.
**USA**	https://www.hrsa.gov/rural-health/about-us/definition/index.html (US census bureau) and the Office of Management and Budget	**The federal government uses two major definitions of ‘rural’**
		a.US census bureau definition: ‘Rural’ encompasses all population, housing, and territory not included within an urban area. There are two types of urban areas: urbanized areas (UAs) of 50,000 or more people; and urban clusters (UCs) of at least 2,500 and less than 50,000 people.
		b. The Office of Management and Budget (OMB) designates counties as metropolitan, micropolitan, or neither. A Metro area contains a core urban area of 50,000 or more populations, and a micro area contains an urban core of at least 10,000 (but less than 50,000) population. All counties that are not part of a Metropolitan Statistical Area (MSA) are considered rural.
		Census data is used by the Federal Office for Rural Health Policy in defining rurality called the Rural-Urban Commuting Area (RUCA) codes. This is to avoid over counting (census bureau definition) or under counting (office of management and budget definition) of rural populations
**United Kingdom**	Office for National Statistics (The Rural Urban Classification, RUC)	Areas are rural if they are outside settlements with more less than 10,000 resident population
**New Zealand**	Statistics New Zealand definitions	Urban areas are defined as those cities and towns with a population of at least 1000 people.
		A detailed classification describes 3 types of urban areas: main urban areas (towns and cities with a minimum population of 30,000 people), secondary urban areas (towns with a population between 10,000 and 29,999 people), and minor urban areas (towns with a population between 1000 and 9999 people) and two types of rural areas: rural centres (population between 300 and 999 people) and true rural areas (population less than 300 people).

Given these definitions differ considerably across geographies, this renders cross-country comparisons problematic. For instance, an area with a population of less than 400 persons in New Zealand is considered rural; whilst an area with less than 10,000 persons in the UK is rural. Population density on its own becomes an empty concept if the context is not specified. Accessibility to services, for instance, healthcare services, also carries some shortcomings. In New Zealand for instance, 40% of people using rural healthcare facilities are classified as urban by Statistics New Zealand greatly contributing to the difficulties in demonstrating the differences between rural and urban healthcare services [17]. The rural-urban dichotomous definitions have been reported to propagate the heterogeneity among rural areas especially when it comes to access to healthcare services [31-34]. Failure to recognize this reality means health policy research recommendations might miss the intended beneficiaries. Inequalities in healthcare, a critical issue in health policy, might persist due to the inability to correctly identify who is a rural or urban resident, as well as correctly identifying the usage patterns of the healthcare services at their disposal. Poor definitions can drive health inequalities since those in need can either be missed/misclassified or received inappropriate healthcare interventions or funding. To mitigate this bias, it is plausible to rethink the definition of 'rural health' that captures the key healthcare challenges or priorities of areas considered as rural. Definitions mainly based on geographical considerations (population density for instance) are inadequate to capture the healthcare needs of rural populations [23].

Identifying a consensus definition for urban areas is also a daunting exercise. In a literature review and expert survey of the European Union member states for the European Urban Health Indicator System (EURO-URHIS) project, no unique definition for urban areas was found [25]. Sociocultural differences between urban and rural areas suggest that residents´ views regarding the meaning of being healthy or quality healthcare might be different. Rural residents have been reported to have distinct views on how they conceptualize and define health [11]. The researchers however highlight in their systematic review the scarcity of corresponding comparative definitions of health from urban residents. Capturing a definition for rural health that is relevant to a specific context stands to benefit from including rural residents´ views. Indeed, whenever possible, and depending on the research subject of interest, co-creating a definition of 'rural' stands to provide findings and policy recommendations that better capture the populations that stands to benefit, as well as reflect the reality of the setting in question. Empirical rural-urban comparative health research is sparse and increasingly being required to provide context relevant healthcare. This is relevant in not only properly ascertaining the real burden of disease in rural and urban areas, but also allows identification of unique healthcare challenges associated with being a rural or urban resident. These high-quality findings can be a strong advocacy tool in pushing policy makers to respond adequately to the needs of the disadvantaged rural residents [24,35].

### The need for an inclusive definition for rural health

#### Theoretical imperative

Bourke and colleagues argue that developing a theoretical framework on how to approach rural health has relevance both at intellectual and policy levels [36]. They put forward five main reasons for this: i) theory provides logical guidance to study a subject; (ii) it helps in challenging assumptions in the knowledge generation process; (iii) permits a structured analysis which is a relevant predictor of knowledge transferability; (iv) theory provides predictability; and (v) theory allows for a holistic understanding of concepts studied. Complexities around the definitions of rural health across the world compel researchers to speak a common language. Theoretically, it is imperative to allow for peers to learn from experiences elsewhere and apply it locally [14]. This is helpful in not repeating the same conceptual and methodological mistakes committed elsewhere. An international rural health research agenda will highlight how rural healthcare provision is different and constitute a great avenue for policy makers to make context specific relevant and appropriate policies.

There is a need for best-practice guidelines to direct researchers on which urban/rural definition is appropriate for which situations. There are cultural differences between rural communities and urban centres in many countries including significant cultural differences as one moves from rural community to the other. Sociologists describe this quality as '*gemeinschaft*' (social relations between individuals, based on close personal and family ties; community) [12,26]. Indeed, through a social lens, the construct of rurality should reflect local understandings, history, lifestyle, and institutions. Most rural populations in the world are from low- and middle-income countries (LMICs) while rural health research centres and researchers are rare in these countries. Most research in rural health has explored rural areas mainly in Western countries, with very limited data on rural health in LMICs.

We can hypothesize that these health policies, generally urban-centric might be neither relevant, nor appropriate, for rural settings. Inequalities in the provision of, and access to healthcare, are prone to persist, or be exacerbated in some instances in these rural settings. The rural populations can be trapped in the vicious cycle of disease and poverty, which negates the core objective of global health (equity in healthcare access) as well as the World Health Organization's (WHO) “Health for All' campaign.

## Conclusion

Worrisome evidence indicates that the gap in mortality rates between rural and urban settings is increasing as the years go by [37]. If ´rural health´ continues to be poorly defined, the over 3.4 billion persons in the world living in these areas (90% in Africa and Asia) might be receiving inadequate and inappropriate healthcare. Indeed, no clear definition of rural areas was found from developing countries. Heterogeneity is a constant reality worth considering in rural health research and policy making. We agree that context remains key when it comes conceptualizing complex subjects like rurality. However, developing minimum criteria to foster communication among rural health researchers is need. Systematically providing operational definitions of what authors describe as “rural” in the rural health research and policy literature is of utmost relevance. With the current trends where rural populations are left behind, attaining the sustainable development goal 10 on health and inequalities remains a mirage [8]. Not all is bad regarding health outcomes when it comes to living in rural areas. Articulating “rurality associated health advantages”, and how these could be enhanced will stand to benefit a huge portion of the world's population which is predominantly “rural”.
